# Pharmacogenomics: Current State-of-the-Art

**DOI:** 10.3390/genes5020430

**Published:** 2014-05-26

**Authors:** Daniel F. Carr, Ana Alfirevic, Munir Pirmohamed

**Affiliations:** Wolfson Centre for Personalised Medicine, Department of Molecular and Clinical Pharmacology, University of Liverpool, Block A Waterhouse Buildings, 1-5 Brownlow Street, Liverpool, L69 3GL, UK; E-Mails: d.carr@liv.ac.uk (D.F.C.); Ana.Alfirevic@liv.ac.uk (A.A.)

**Keywords:** pharmacogenetics, personalized medicine, pre-treatment genetic testing

## Abstract

The completion of the human genome project 10 years ago was met with great optimism for improving drug therapy through personalized medicine approaches, with the anticipation that an era of genotype-guided patient prescribing was imminent. To some extent this has come to pass and a number of key pharmacogenomics markers of inter-individual drug response, for both safety and efficacy, have been identified and subsequently been adopted in clinical practice as pre-treatment genetic tests. However, the universal application of genetics in treatment guidance is still a long way off. This review will highlight important pharmacogenomic discoveries which have been facilitated by the human genome project and other milestone projects such as the International HapMap and 1000 genomes, and by the continued development of genotyping and sequencing technologies, including rapid point of care pre-treatment genetic testing. However, there are still many challenges to implementation for the many other reported biomarkers which continue to languish within the discovery phase. As technology advances over the next 10 years, and the costs fall, the field will see larger genetic data sets, including affordable whole genome sequences, which will, it is hoped, improve patient outcomes through better diagnostic, prognostic and predictive biomarkers.

## 1. Introduction

The field of pharmacogenomics can trace its roots back to significantly earlier than the first draft publication human genome sequence in 2001 [1] and the subsequent completion in 2003. Indeed, the term “Pharmacogenetics” was first coined by Friedrich Vogel in 1959 [2], just 6 years after Watson and Crick’s discovery of the structure of DNA [3].

Though significant progress has been made in the field since 2003, it could be argued that pharmacogenomics has failed to live up to expectations. A vast number of discoveries relating to genomic variability and drug response have been made in the last 10 years. The challenge remains to translate these findings into clinical practice for the benefit of the patient.

## 2. The International HapMap Project

The completion of the first phase of the International Hapmap project [4], a catalogue of common genetic variations within individuals of diverse ethnicities, in 2003, provided a rich data resource which enabled researchers to investigate the association of variants across the human genome with a wide range of clinical phenotypes. Indeed, the data derived from this allowed the creation of SNP arrays whereby researchers could analyze patient genotype for 100,000 s to millions of SNPs at a time. Thus, for the first time, unbiased genetic analysis of clinical phenotype/genotype associations was possible using platforms created for the Genome Wide Association Study (GWAS). More recent advances in genomics have seen the development of next generation sequencing methodologies which do not require *a priori* knowledge of genetic variation.

Mining of the National Human Genome Institute (NHGRI) genome wide association study (GWAS) catalogue [5] (accessed on 22nd April 2014) showed that a total of 1885 peer-reviewed articles have been published reporting GWAS findings. However, only 93 (4.9%) are related to inter-individual variability of drug response phenotypes (either safety or efficacy).

Despite the small numbers of studies to date, which have investigated common genetic variant associations with pharmacological phenotypes, a number of important genotype-phenotype associations have been identified in pharmacogenomics using GWAS approaches (Table 1). The interesting phenomenon, when compared with GWAS of complex diseases, is that the effect size for pharmacogenomics phenotypes on the whole seems to be larger than that observed for complex diseases, which allows for (a) smaller sample sizes to be studied, which is more time- and cost-efficient, and (b) some variants to be considered for clinical implementation in terms of use prior to drug prescription, which contrasts with complex diseases, where the relative risks identified are usually below 1.5 and have not been used clinically. Whilst common genetic variation has been shown, in a number of examples to be important factors determining inter-individual variability in drug response, the role of rare, or private, variants is unclear in pharmacogenomics phenotypes, and is an important area for further research.

**Table 1:** Genetic biomarkers of (**A**) adverse drug reactions and (**B**) inter-individual variability of drug efficacy identified, or confirmed, from genome wide association studies identifying.

A	Year	Drug	Indication	Phenotype	Population	Associated Loci	SNP/Allele	Ref.
	2008	Simvastatin	Hypercholesterolaemia	Myopathy	Caucasian	SLCO1B1	rs4149056 (c.521T>C/*5)	[6]
	2008	Bisphosphonate	Multiple Myeloma	Osteonecrosis of the jaw	Spanish	CYP2C8	rs1934951	[7]
	2008	Ximelagatran	Anticoagulant	Hepatotoxicity	Caucasian	HLA-DRB1	*07 and *02	[8]
	2009	Flucloxacillin	Macrolide antibiotic	Hepatotoxicity	Caucasian	HLA-B	B*57:01	[9]
	2011	Carbamazepine	Epilepsy	Skin Rash/Hypersensitivity	Japanese Causasian	HLA-A	A*31:01	[10,11]
	2013	Allopurinol	Gout	SJS-TEN	Japanese	HLA-B	B*58:01	[12]
**B**	**Year**	**Drug**	**Indication**	**Phenotype**	**Population**	**Gene Loci**	**Allele**	**Ref** **.**
	2009	Clopidogrel	Antiplatelet		Amish	CYP2C19	*2	[13]
	2009	Pegylated Interferon	Hepatitis C		Caucasian	*IFNL3* (IL-28B)	rs12979860	[14]
	2009	Warfarin	Anticoagulant		Caucasian	CYP2C9 and VKORC1	*2,*3/c.-1639G>A	[15]

## 3. The 1000 Genomes Project

The first findings from the 1000 genomes project were reported in 2010 [16]. This has provided researchers with a population scale map of rare variants to complement and enrich existing knowledge of common variants gained from the HapMap project. With the per-base cost of sequencing using “next generation sequencing (NGS)” platforms continuing to fall, it is likely that studies will further investigate the role of rare genetic variants in defining variation in drug response phenotypes.

A recent study reported the mapping of rare and common variants within 12 Cytochrome P450 genes, thought to be responsible for metabolizing 75% of prescribed drugs [17]. Using whole exome sequence data for 2203 African Americans and 4300 Caucasians, researchers were able to identify novel, potentially deleterious alleles in major drug metabolizing enzymes in 7.6%–11.7% of individuals. The power of NGS technologies to identify rare variants could allow for greater understanding of their contribution to inter-individual variability in drug responses where common variants explain a limited degree of variability. One such example where rare variants may enhance our understanding of variability is the case of warfarin dose prediction. Incorporating CYP2C9*2, *3 and VKORC1 polymorphisms along with clinical confounding factors into a dosing algorithm allows clinicians to predict ~60% [18] of dose variability. However, it is entirely plausible that incorporating the contribution of rare functional genetic variants into such algorithms may in the future allow for even greater accuracy in warfarin dose prediction. Indeed a number of small scale studies and case reports have already identified rare missense variants in the VKORC1 gene in warfarin resistant patients [19,20,21,22]. Another example is drug-induced torsades de pointes where at least 10% of cases of may be due to rare mutations in the congenital long QT syndrome genes [23]. At least 23% of Caucasian subjects with drug-induced torsades de pointes carry a variant within 22 congenital arrhythmia genes (which include the 13 congenital long QT syndrome genes), compared with a background rate of 1.7% in 60 control subjects from the 1000 Genomes CEU data [24]. As greater numbers of NGS-based analyses in pharmacogenetic studies are undertaken, so the contribution of rare variants in other drug safety and efficacy phenotypes will be better understood.

## 4. Non-Coding RNAs

Large-scale, coordinated, international, research efforts, such as the ENCODE project [25] have expelled the myth that large regions of our human genome are “junk” DNA. Indeed, the presence of non-coding RNAs has altered the scientific community’s perception of the central dogma of molecular biology. To date, only a very small number of studies have investigated the application of small non-coding RNA molecules as biomarkers of variable drug response.

However, studies have shown a potential utility for specific microRNAs as markers of both drug-induced liver injury (miR-122 and -192) [26] and severe skin reactions (miR-18a-5p) [27]. Though such biomarkers are still at the discovery stage, further clinical validation may see these and other non-coding RNAs enter clinical practice as early-stage pharmacogenomics predictors of adverse drug reactions.

## 5. Clinical Utilization of Pharmacogenomics

The biggest challenge that has faced the field of pharmacogenomics in the 10 years since the completion of the human genome project is clearly the application of genetic markers of variable drug response to decision-making in relation to prescribing. Indeed, as recently as 2011 it has been estimated that, of the >150,000 papers reporting claimed biomarkers, less than 100 has made it into clinical utility [28]. This of course refers to all biomarkers, not just pharmacogenomic biomarkers; whether pharmacogenomics biomarkers have been more successful is unclear, and would require formal analysis.

There are currently 121 Food and Drug Administation (FDA) drug labels referring to pharmacogenetic biomarkers of drug safety or efficacy [29]. Only a very small proportion of these drug labels mandate clinicians to test for pharmacogenetic markers (e.g., abacavir and HLA-B*57:01; and carbamazepine and HLA-B*15:02 in Southern Asians). However, a large number of testing guideline position papers have been published by the Clinical Pharmacogenetics Implementation Consortium [30]. The aim of this collaborative effort is to help clinicians understand how genetic tests may be used to guide treatment decisions.

One reason why many pharmacogenetic biomarkers have failed to move from discovery to clinical implementation is that many genotype/phenotype associations fail to be independently replicated. It has been recognized that variability in phenotype definition could contribute to this, particularly in relation to adverse drug reactions. To this end a number of phenotype standardization efforts have been undertaken in recent years. These include drug-induced skin injury [31], liver injury [32] and Torsade de Pointes (long-QT syndrome) [33]. It is hoped that, with studies applying consistent phenotype definitions, future pharmacogenetic studies may identify replicable pharmacogenetic biomarkers that, with sufficient weight of evidence, could find their way into clinical utility. Another reason for lack of replication is the inability to find replication sample sets, particularly where the phenotype is a rare adverse event. In such cases, functional genomic analyses may reduce false positives, and provide more confidence for implementation because of insights into the mechanism of effect of that biomarker.

## 6. The Future…

### 6.1. Point of Care Genetic Testing

As our understanding of the human genome has grown, so the technology with which we can analyze it has developed in terms of speed and fidelity. The polymerase chain reaction, first described by Kary Mullis in 1983 [34], allowed sensitive analysis of DNA and ultimately yielded a number of pharmacogenetic biomarkers in the pre-genome era based on prior knowledge of gene function (candidate gene studies). As with our understanding of the genome, PCR-based technology has advanced significantly and now allows for rapid and accurate genotype detection. In recent years a number of studies have investigated the potential utility of rapid point-of-care (POC) genetic testing. 

Two key randomized control trials have recently utilized pre-treatment genetic testing, with POC devices, to guide the use of drugs used in cardiovascular medicine, the anticoagulant coumarin derivatives (e.g., warfarin) [35] and the antiplatelet drug clopidogrel [36]. The EU-PACT study [35] randomized patients to either genotype-guided or standard dosing. The genotype guided group, utilized molecular beacon technology to genotype for the CYP2C9*2 and CYP2C9*3 variants and a promoter polymorphism of *VKORC1* prior to the patient receiving warfarin. The results generated were then incorporated into a warfarin dose calculator, incorporating a number of key non-genetic factors affecting dose requirement. The warfarin trial found that patients randomized to pharmacogenetic-guided dosing spent a mean percentage time in the therapeutic range of 67.4% compared with 60.3% on the standard dosing protocol. In this example, the healthcare professional was presented with an individualized, genotype-guided dosing regimen for the patient based on not only clinical variables but also genetic factors. The technology used in the EU-PACT trial had a lead time of approximately 2 h to obtain the required genotypes.

In the case of the RAPID-GENE trial [36], patients were typed for the CYP2C19*2 allele, which has been associated with a lack of efficacy of clopidogrel following percutaneous coronary intervention (PCI). Patients were randomized to standard (75 mg/day clopidogrel) or genotype-guided arms. In the genotype guided arm, patients carrying the CYP2C19*2 allele were prescribed 10 mg prasugrel while wild-type patients were given the standard therapy of 75 mg/day clopidogrel. The trial demonstrated that genotype-guided dosing significantly reduced adverse events (platelet reactivity) compared to standard treatment. The genotyping technology utilized for RAPID-GENE, the Spartan RX CYP2C19, is able to produce genotypes in less than 60 min. In August 2013, the Spartan RX CYP2C19 device became the first on-demand rapid genetic testing device for prescribing guidance to gain Food and Drug Administration (FDA) approval. However, it is important to note that it is not approved as a POC device and must be operated within a clinical laboratory environment.

POC Genotype technologies continue to advance and molecular biology methodologies, such as SmartAmp 2 [37] are reducing the time to obtain a genotype further. It is already possible to determine genotypes for pharmacogenomics-related variants such as those associated with warfarin dose requirement (*VKORC1* and *CYP2C9*) in 30–40 min [37,38]. With addition of microfluidics technology to devices [39,40], the size of the equipment for genotyping will decrease and subsequently, the portability will also increase. It is the ease of use and interpretation of results which are the key attributes for POC genetic test which will facilitate their adoption by health-care professionals. In future years, it is likely that many more devices and tests will be applied to clinical care and obtain regulatory approval.

### 6.2. Companion Diagnostics

For a number of years now the notion of “one size fits all” in both drug prescribing and drug development has seemed an outdated concept. Indeed, the pharmaceutical industry has re-focused its efforts away from the previous block buster drug model to develop more “niche-busting” products which are licensed alongside a companion diagnostic assay (Table 2). This allows for drugs which may be largely considered ineffective in the wider population to be targeted to a subset of patients likely to respond well to the treatment. To date, the vast majority of these have been developed in the oncology field but it is likely that many more examples of population stratification using genomics methodologies for targeted treatment will emerge for other indications. An added advantage of undertaking targeted therapies in cancer using companion diagnostics has been the rapid approvals obtained from the FDA, despite pivotal efficacy trials testing smaller numbers than is usual in non-stratified trials [41].

While the majority of companion diagnostics products to date have focused on variation in pharmacodynamic factors for stratification, there is also a growing interest in individualizing drug doses based on pharmacokinetic variability. For example, dose escalation of tivantinib, a non-small cell lung cancer therapy, is based on stratification for the *CYP2C19* genotype [42]. This is consistent with European Medicines Agency guidance on pharmacogenetic effects on drug pharmacokinetics [43]. Thus, in the future, it is likely that regulatory approval for a new drug could be dependent on dose stratification based on the underlying metabolizer phenotypes. 

### 6.3. Pre-Emptive Genotyping

The possibility of pre-emptying genotyping is being explored in the U.S., for example, through the Vanderbilt Personalized Medicines Program, where patients are genotyped on a 184-variant platform [44]. The theory behind this approach is that if a genotype is available to the physician in the electronic medical record, at the point of prescribing, they will be able to make a rational decision as to the choice and/or dose of the drug. It also highlights an important issue in relation to the evidence for clinical implementation: we cannot possibly undertake randomised controlled trials or prospective studies for every genomic variant that is identified, and other methodologies for evaluating the clinical utility of a genomic biomarker will have to be utilized. It is also important to note that such information is already used by physicians with respect to non-pharmacogenomic tests, for example renal function tests allow clinicians to reduce the dose of a drug that is renally excreted [45]. Whether and how such pre-prescription genotyping will be used by prescribers, and whether it leads to improvement in clinical outcomes, will require careful evaluation over the next few years. Indeed it can be seen as a prelude to a time when whole genome sequencing is so cheap that is undertaken routinely with the data being available within electronic medical records.

### 6.4. Personal Genomes and Clinical Applications

The per-base cost of sequencing has fallen exponentially since 2003 and has for many years exceeded the trajectory of Moores Law (the number of transistors on integrated circuits approximately doubles every 2 years) [46]. With this is mind it is feasible to imagine in the not too distant future that whole genome sequencing will be a cost effective option for healthcare providers. However, with the >3 billion base pairs confirmed in 2003 by the human genome project and the complexity of interpreting the role of genetic variation in inter-individual drug response, it is likely the challenge for the next 10 years will be in producing the tools with which to interpret this data and provide meaningful outputs that can be utilized by healthcare professionals.

**Table 2:** *In vitro* companion diagnostic kits currently licensed by the Food and Drug Administration (FDA).

Biomarker	Prevalance	Indication	Drug	Assay Kit	Technology	Manufacturer
HER2 gene amplification	22.2% [47]	Breast cancer	trastuzumab (Herceptin)	Inform	FISH	Ventana Medical Systems
				PathVysion #	FISH	Abbott Molecular Inc.
				SPOT-Light HER2	CISH	Life Technologies
				InSite HER2	IHC	Biogenex Laboratories, Inc.
				HercepTest	IHC	Dako Denmark
				HER2 PharmDx	CISH	Dako Denmark
				HER2 PharmDx *	FISH	Dako Denmark
				PATHWAY Her2	IHC	Ventana Medical Systems
				Bond Oracle	IHC	Leica Biosystems
EGFR protein expression	60%–80% [48]	Colorectal cancer	cetuximab (Erbitux) panitumumab (Vectibix)	EGFR pharmDx	IHC	Dako North America
c-Kit protein expression	100% [49]	Gastrointestinal stromal tumours	imatinib (Gleevac)	c-kit pharmDx	IHC	Dako North America
*ALK* gene rearrangement	7.5% [50]	Non-small cell lung cancer	crizotinib (Xalkori)	VYSIS	FISH	Abbott Molecular Inc.
*BRAF* p.V600E mutation	75.4% [51]	Melanoma	vemurafenib (Zelboraf)	Cobas 4800	Real-time PCR	Roche Molecular Systems, Inc
*KRAS* mutation	30%–60% [52]	Colorectal cancer	cetuximb (Erbitux)	therascreen	Real-time PCR	Qiagen

* Indicated for assessment of breast cancer patients considered for pertuzumab (Perjeta) or # cyclophosphamide, doxorubicin, 5-FU treatment.

### 6.5. Ethical Considerations

A key ethical issue with regard to the implementation of personalised medicine relates to the fact that it may lead to health inequalities, within and between countries. Given the costs related to developing, manufacturing and obtaining approval for genetic tests, in addition to defining the role of ethnic genetic differences, it is easy to see how resource poor countries and communities could miss out on the benefits of personalised medicine advances in the future. This may be offset by the advances in genomics technologies and the vast reduction in costs of implementing them that has taken place over the last 10 years. Another issue which is becoming increasingly important with the use of next generation sequencing technologies is whether patients should be informed about incidental findings. An analysis of 1000 participants’ exomes showed that the frequency of actionable (*i.e.*, pathogenic or likely pathogenic single nucleotide variants) incidental findings was 3.4% in European Caucasians and 1.2% in Africans [53]. Some guidelines have been produced [54], but there is still controversy [54,55], and the debate will no doubt become more intense as more people have their genomes sequenced, and more variants are classified as being actionable.

### 6.6. Educating Stakeholders

In order for pharmacogenomics, as one of the technologies that is important for personalised medicine, to realize wider uptake into healthcare provision, there needs to be greater awareness and education. This applies not only to healthcare professional but to patients who are the ultimate stakeholder in pharmacogenomics, and stand to gain the most if we can improve predictability of how patients will respond to drugs.

Clinicians need to be made aware of the availability of genetic tests relating to treatment decisions, and how to interpret them. Lack of familiarity with genetic tests may be one reason for the poor uptake into clinical practice [45]. The lack of training is amongst the most common reasons cited as a barrier to pharmacogenomic implementation [56].

### 6.7. Regulatory Environment

There are of course many other issues which need to be tackled in order to facilitate the implementation of pharmacogenomic testing into clinical practice—many of these also apply to other biomarker strategies that are important for personalised medicine [41]. Amongst this is the regulatory environment, which has a big influence on the diagnostics industry. With the development of companion diagnostics, it will be important for there to be streamlined procedures which allow for simultaneous approval of the drug and diagnostic. There are differences between the FDA and European Medicines Agency for the development of companion diagnostics, with the latter being less stringent, but likely to adopt similar procedures in a drive to ensure there is global harmonization of the regulatory procedures needed for approval [57]. For the diagnostics industry, the requirement for a clinical utility study may become prohibitive in terms of cost, unless there are clear pathways for protection of intellectual property and re-imbursement. 

## 7. Conclusions

Since the completion of the human genome, there has been steady, albeit slow, progress in the identification and implementation of biomarkers into clinical practice. This progress is likely to continue, and hopefully accelerate as our ability to interrogate the human genome becomes more cost- and time-efficient, and we start embracing, and intelligently interpreting, different sources of data to define the clinical validity and utility of biomarkers. Outcomes research will thus become particularly important, and will depend on having access to curated electronic healthcare databases where patients can be followed longitudinally from the time of having a biomarker assessed to the time a drug is prescribed, and forward into the future to define the clinical outcome of the patient (in comparison to relevant *a priori* defined controls).
